# Gastrostomy in Children: A 5-Year Single Tertiary Center Experience

**DOI:** 10.3390/medicina61030459

**Published:** 2025-03-06

**Authors:** Iulia Florentina Ţincu, Bianca Teodora Chenescu, Gabriel Cristian Drăgan, Anca Ioana Avram, Doina Anca Pleșca

**Affiliations:** 1 Faculty of Medicine, Pediatrics Department, “Carol Davila” University of Medicine and Pharmacy, 020021 Bucharest, Romania; iulia.tincu@umfcd.ro (I.F.Ţ.);; 2 “Dr. Victor Gomoiu” Clinical Children Hospital, 022102 Bucharest, Romania

**Keywords:** gastrostomy, SAGA-8, laparoscopic-assisted gastrostomy

## Abstract

*Background and Objectives*: Pediatric patients with complex medical conditions, including neurological impairments, genetic syndromes, dysphagia, and malnutrition, often face feeding difficulties that require enteral nutrition support. The optimal technique for gastrostomy tube (GT) placement in children remains unclear, with options such as laparoscopic gastrostomy and percutaneous endoscopic gastrostomy (PEG) being compared in previous studies. This study evaluates outcomes, including complications and caregiver satisfaction, associated with different GT placement techniques in pediatric patients, focusing on the impact of concomitant anti-reflux surgery (fundoplication). *Materials and Methods*: This retrospective analysis of 71 children (34 with anti-reflux surgery [Group 1], 37 without [Group 2]) undergoing GT placement between 2019 and 2024. Data included demographics, procedural details, complications, and caregiver satisfaction assessed via the Structured Satisfaction Questionnaire with Gastrostomy Feeding (SAGA-8). *Results:* A total of 71 patients (34 in Group 1, 37 in Group 2) were included in the final analysis. The mean age at the time of the procedure was 5.0 ± 1.1 years, with cerebral palsy being the most common underlying condition. Laparoscopic GT was performed in 97% of cases (69/71), with two percutaneous endoscopic gastrostomy (PEG) placements. Common complications included suppuration (32.35% Group 1 vs. 21.62% Group 2, *p* = 0.88) and infection (5.88% vs. 2.70%, *p* = 0.67). There were no significant differences between groups in terms of complication rates, although patients in Group 1 had longer hospitalization durations (7.51 ± 3.56 days vs. 4.22 ± 2.13 days, *p* < 0.005). Caregiver satisfaction, as assessed by the SAGA-8, was high, with 84.5% of families reporting positive outcomes. Factors influencing satisfaction included previous aspiration pneumonia and the use of home blenderized diets post-discharge. *Conclusions:* Both laparoscopic and PEG techniques are associated with low complication rates and high caregiver satisfaction in pediatric patients requiring gastrostomy placement. The laparoscopic approach may be preferred for patients undergoing concomitant fundoplication.

## 1. Introduction

Pediatric patients with complex underlying medical issues and comorbidities, like neurological impairment genetic syndromes, oro-motor dysfunction, eating disorders, dysphagia, malabsorption, or maldigestion, encounter feeding difficulties as one of the prognostic factors [1]. The actual recommendation of the European Society of Pediatric Gastroenterology Hepatology and Nutrition (ESPGHAN) refers to the need for enteral support nutrition in order to avoid malnutrition in chronic severe diseases using a percutaneous endoscopic gastrostomy (PEG) whenever non-oral nutritional support is anticipated to be required for a period of longer than 3–6 weeks or when trans-nasal tube feeding is unsafe [2]. There has been a shift regarding gastrostomy tube (GT) positioning technique in children in recent decades, which has undergone a transition from classical to laparoscopic procedures and then to percutaneous endoscopic gastrostomy (PEG) insertion. Nevertheless, the best method of gastrostomy placement in children is currently unknown.

Family perspective after gastrostomy placement is important because the decision making is always complex [3], and usually emotional, physical, medical, and resource criteria are also taken into consideration by the families [4]. Initiating gastrostomy feeding is unusually accompanied by positive effects but also challenging considerations that impact the interaction between family and healthcare providers [5]. There is a growing need to use tools that can assess nutritional intervention benefits in relation to symptoms after gastrostomy in pediatric populations in order to improve decision making. After performing a gastrostomy, it is important to evaluate the quality of life (QoL) and satisfaction of caregivers; studies on this issue ended up showing both positive and negative results affected by socioeconomic status, family beliefs, and social values [6].

Many studies have compared the effectiveness and safety of the available techniques in terms of postoperative complications. Caregivers need to be aware of various complications when taking care of a child with gastrostomy; sometimes, a decrease in life quality is related to feeding after GT insertion due to necessary devices for safe and efficient nutritional support [7]. The degree of satisfaction after GT insertion is not very well studied in the pediatric population. This assessment is not always easy because patients are often too young and/or suffer from serious neurological diseases that prevent them from adequately responding to questionnaires. There is a need for satisfaction assessment by care providers, even indirectly [8].

The present research was aimed at analyzing the degree of satisfaction in patients receiving GT using a Structured Satisfaction Questionnaire with Gastrostomy Feeding. We were also interested in evaluating the characteristics of patients and the techniques used for the positioning of the gastrostomy in relation to outcomes.

## 2. Materials and Methods

### 2.1. Study Design and Population

This was a retrospective study aiming to analyze cases that received gastrostomy placement from January 2019 to January 2024 in “Dr. Victor Gomoiu” Clinical Children Hospital, in Bucharest, Romania. Inclusion criteria were age under 18 years old and need for gastrostomy insertion; patients not following the study procedures or lost for surveillance were excluded. All clinical and laboratory data were collected from the hospital’s database. Patients requiring gastrostomy placement, regardless of indication and technique, were enrolled in the study. For outcome analysis, individuals were divided into 2 groups: patients receiving anti-reflux surgery (Group 1) and patients who did not require anti-reflux surgery (Group 2). We excluded from the final analysis individuals who already received gastrostomy by the time they were admitted to the hospital.

### 2.2. Data Collection and Procedures

Data collection was made using a specific observational yes/no answer sheet and included information on four categories: (1) general information: age at diagnosis (years), gender (male/female), living area (rural/urban), underlying condition (cerebral palsy, multiple sclerosis, feeding disorder), gastrostomy indication (GERD, malnutrition, aspiration, dysphagia), preexisting genetic syndromes, aspiration pneumonia; (2) anthropometry assets before the intervention and every following visit: weight, height, Z score for body mass index at diagnosis; (3) feeding practices before and after the procedure: previous feeding procedure (oral, naso-gastric tubes, mixed), types of food used before GT (standard formula, enteral formula, normal diet), types of food used after GT (formula feeding, home blenderized diet, modular diet), meal duration in hours, time for the first feeding in hours, usage of night continuous nutrition; (4) procedure information: technique (laparoscopic, endoscopic); type of GT (long/short profile), number of hospitalization days, symptoms (nausea, eructation, reflux, vomiting, flatulence, abdominal pain, constipation, diarrhea, mouth dryness); complications (suppuration, infection, disjunction, fistula, bumper retraction); satisfaction index—we used Structured Satisfaction Questionnaire with Gastrostomy Feeding (SAGA-8), in order to assess the degree of satisfaction while using GT. This is specifically intended for parents and caregivers of children who receive enteral nutrition using GT [7], validated in previous studies [9]. The questionnaire takes into consideration eight issues, with 1 to 5 satisfactory answers, ranging from 1 (totally unsatisfied) to 5 (very satisfied): (Q1) parents’ acceptance towards gastrostomy; (Q2) parents’ evaluation of GT management; (Q3) caregivers were asked to evaluate the support offered by our center; (Q4) opinion upon the child nutritional status; (Q5) the change in their child and your family’s overall situation; (Q6) decrease in time feeding; (Q7) decreased in respiratory infections; (Q8) parents were asked if they would accept earlier GT placement with your current knowledge of the procedure’s benefits. Accordingly, the total score of SAGA-8 ranged from 8 to 31 points [9].

The team also included pediatric surgeons who evaluated patients before the procedure. Regardless of the insertion technique, all the patients underwent a contrast radiographic examination. Concomitantly, caregivers were advised to accept pH and impedance studies (pH-MII) to assess underlying gastroesophageal reflux disease (GERD). The analysis was considered positive for GERD by the symptom index higher than 50% (SI; number of symptoms associated with reflux/number of all symptoms × 100) and the total number of reflux episodes over 70 episodes in 24 h in patients aged >1 year and >100 episodes in those aged <1 year [10].

Subjects identified with GERD were proposed laparoscopic fundoplication and laparo-assisted gastrostomy [11]; we used PEG for endoscopic GT (the pull-through technique with a mushroom probe). During the endoscopic procedure, we confirmed the PEG position by positive transillumination through the stomach and abdominal wall. Perioperative antibiotic prophylaxis was used in all cases.

The data were collected at the placement of the gastrostomy (T0), and the follow-up procedure included scheduled visits at 1 month (T1), after 3 (T3), and 6 months (T4) from insertion, except for the cases evolving with complications.

### 2.3. Primary and Secondary Endpoints

The primary outcome consisted of the percentage of patients suffering from complications and, secondly, the effect on quality of life assessed by the SAGA-8 questionnaire in the two groups.

### 2.4. Ethics

The study design adhered to the ethical principles of the Declaration of Helsinki and was approved by the ethics committee of the “Dr. Victor Gomoiu” Clinical Children Hospital (no 9215/9 June 2023). Written informed consent was waived from all the enrolled patients’ caregivers due to the retrospective characteristic of the study.

### 2.5. Statistical Analysis

Analyses were performed with the SPSS 23 software package (SPSS Inc., Chicago, IL, USA). All demographic and clinical variables were summarized using count and percentage *n* (%) for categorical variables and means plus or minus standard deviations for continuous variables. Continuous variables were tested for normality of data distribution, and normally distributed data were expressed as mean and SD. In order to compare categorical variables when appropriate, the authors used the Fisher exact test and Pearson X2 tests, and for continuous variables, the student’s *t*-test was used for comparison. *p* values < 0.05 were considered statistically significant.

## 3. Results

In the study period, namely January 2019–January 2024, 76 gastrostomies were placed in our center, but 5 had incomplete data, so the final analysis refers to the remaining 71 subjects, 34 for Group 1 and 37 for Group 2. Out of these, 34 were female (47.88%), and 37 were male (52.11%). The median age at the time of the procedure was 5.27 ± 1.19 years, ranging from 2 months to 17 years for the entire cohort, but group analysis is shown in Table 1. Cerebral palsy was the most common associated condition for both groups. The main indication for GT was a combination of malnutrition and dysphagia, and the rest of the indications are shown in Table 1. Data were not stratified according to age.

There were only 2 patients receiving PEG (based on parental acceptance), so most of the others had a long profile GT with no balloon. The medium hospitalization duration involving the procedure was 7.51 ± 3.56 and 4.22 ± 2.13 for Groups 1 and 2, respectively. This was mainly explained by the fact that patients in Group 2 underwent the procedure during a longer hospital stay due to their neurologic illness. The main symptom after insertion was abdominal discomfort for both groups. There was no difference between the two groups in terms of complication rate. Suppuration was the most commonly encountered complication of GT (Table 2).

The results of using the SAGA-8 score are detailed in Table 3. A high satisfaction rate was reported by 84.5% of the families (*n* = 60). Most parents/caregivers agreed with the feasibility of the procedure (90.14%, *n* = 64).

**Figure 1:**
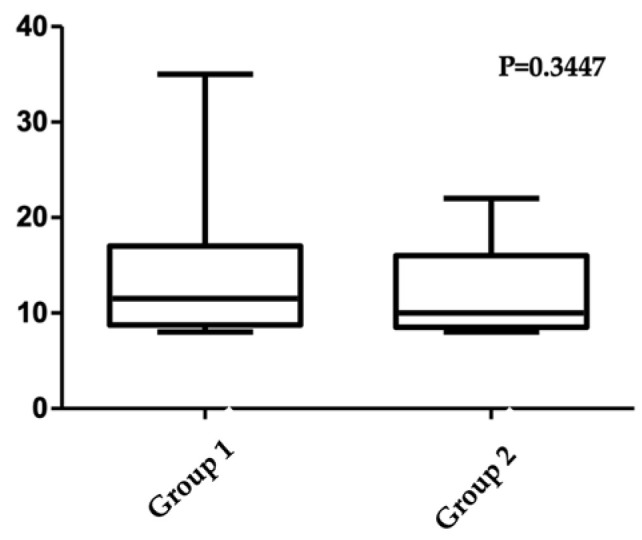
SAGA-8 variation in the study groups.

SAGA-8 was similar for both working groups, regardless of fundoplication indication; similarly, a longer duration of hospitalization was not associated with lower satisfaction scores (Figure 1). We did not apply for any cofounders. Meanwhile, patients who previously experienced aspiration pneumonia were more satisfied after the procedure, as well as families that adopted a home-blenderized diet after discharge.

## 4. Discussion

Considering local literature in our country, this is the first research aiming to analyze the degree of satisfaction in pediatric patients requiring gastrostomy, according to the technique that was used. This study included a large age range in the pediatric population, aiming to compare indications and outcomes when divided by the need for a concomitant fundoplication procedure. Whenever nutritional support is required for a longer period of time, meaning over 3 months, there is an indication for using gastrostomies in children [12]. Over the last decade, PEG has become the preferred technique, mainly in adults, having fewer complications than surgical insertion.

More than 65% of the subjects, according to the z-score of BMI, had disease-associated malnutrition at the time of gastrostomy insertion, and the main indication of an underlying condition was central nervous system disorders or neuro-muscular conditions. Similar data were reported by Jeličić Kadić et al. [13] in a cohort of Croatian children over 11 years [14].

Some studies have previously compared the outcomes of PEG and GT, including data from larger meta-analyses. One paper reviewed 22 studies gathering 5438 patients and declared that GT laparoscopically placed had a significantly decreased risk of major complications when compared to PEG, mostly because visceral injury appeared to be more prevalent [15]. In 2018, Sandberg et al. showed in a systematic review containing 1550 patients from eight studies that the odds of major complications were more than threefold higher with PEG (5.4%) compared to laparoscopic gastrostomy (1.0%) [16]. Similar findings are discussed in another systematic review and meta-analysis including five retrospective studies that declared similar completion rates and minor complications between PEG (550 patients) placements and laparoscopic gastrostomy placements (483 patients), but the first group required more frequent intervention under general anesthesia for bowel injuries, and early tube dislodgement [17]. The review published by Salazar et al. evaluating variability in the method of gastrostomy placement in children in the United States of America over a large period of time, meaning from 1997 to 2012, with 67,811 patients included, declared that national distribution of PEG vs. GT has remained relatively stable over time. Principle variables considering the method of placement vary according to patient age, insurance type, hospital location, and hospital type [18].

The study is similar in age distribution and indications with other cohorts. The mean age at the moment of gastrostomy was 4.86 ± 1.09 and 5.15 ± 1.11 years, respectively, in the two study groups, ranging from 2 months to 17 years; the mean values seem to be higher than previously cited in the literature [19] and lower than in other cohorts [13], but large variation is known to be dependent on indications and referral centers.

The largest group of patients in terms of medical indication is formed by neurologically impaired subjects, who have a high risk of nutrition imbalance over time; according to actual guidelines and recent reviews, gastrostomy placement may be more effective and safer compared with a naso-gastric tube for long-time outcomes [20,21].

In this cohort, we applied methods of clinical and procedural investigations considering associated GERD in order to indicate surgical therapy, and this is the procedure used in similar centers [22]. There is a lack of recommendations regarding routinely evaluating for GERD in children proposed for GT placement [23,24], even so, we performed contract upper gastrointestinal X-ray studies in all patients, pH-MII monitoring in 17 subjects, and endoscopy in 52 children; almost 50.70% of our patients concomitantly had GERD, and fundoplication was performed in 34 (47.88%) patients. The high prevalence of the neurological patients associated GERD in our cohort explains the rate of fundoplication; the same data are reported by Bawazir et al., considering that malignancy in the upper gastrointestinal tract is less frequent in the pediatric population [25].

There was no mortality related to gastrostomy in our individuals, maybe because urgent gastrostomy in the Intensive Care Unit was not applied in our cohort, although literature data indicated 19% death in similar cohorts, even so not related directly to gastrostomy procedures [13]. Placement of a gastrostomy tube is a widely used method for nutritional recovery or in neurologically impaired children, but it is not free of possible complications. Nevertheless, the rate of serious adverse events is low, but some damaging complications were noted, like esophageal fistula due to bumper migration [26]; even so, this is more frequently seen in adult patients [27].

In terms of technique, the laparoscopic procedure was used in the vast majority of our cases; a recent survey indicated no difference in terms of safety and complications considerations when comparing open versus laparoscopic and open versus percutaneous-endoscopic technique [28]. We could not see any complications, regardless of technique, requiring ongoing procedures under general anesthesia with oro-tracheal intubation, that could enhance the risk for aspiration pneumonia preventable by special devices [29]. Since there is an ongoing debate in terms of risk for complications depending on placement technique, further studies are needed in order to establish the most appropriate intervention in pediatric patients.

In our cohort, various types of enteral formulae were used, depending on parent preferences and after-discharge family resources. This is why, at least for this moment of the analysis, we did not remark on terms of malnutrition recovery.

The low burden of gastrointestinal symptoms after gastrostomy placement was maintained across the follow-up period of this study, and these outcomes are also mentioned in some previous reports [30].

There is an important burden on children with home enteral nutrition after GT insertion in terms of family implication and effort. Mainly, this population is unable most of the time to express its degree of quality of life, so practitioners rely on parents’ reports, considering that any procedure success is markedly influenced by the caregiver’s acceptance and home enteral nutritional improvement. SAGA-8 was previously used in order to detect early the degree of satisfaction in families with children requiring GT placement so professionals can lead a better attitude towards those cases [9]. Our experience showed that placing GT ended in a low rate of major complications and an average of high familial and patient satisfaction. Nearly 85% (*n* = 60) of families reported high values for satisfaction questionnaire with gastrostomy feeding, but inconsistent results are presented in the literature; for example, Franken et al. [31] found that health-related QoL was significantly lower in neurologically impaired children after gastrostomy than in neurologically normal children; on the other hand, papers like Sumritsopak et al. [32] and Jennuvat et al. reported an increase in overall satisfaction [33].

This research has some limitations that we are aware of. Due to its single-center retrospective characteristics and an important number of patients with incomplete data or lost from follow-up, recruited cases and clinical management are some of the confounding variables. We consider a strength point of our study to be that this is, to the best of our knowledge, one of the few researches in our country considering gastrostomy in children involving complex data in terms of indication, nutritional status, procedure considerations, and family satisfaction.

## 5. Conclusions

Based on the literature and our own data, the laparo-assisted technique is the best choice for patients who require laparoscopic fundoplication, mainly in neurologically impaired children, and the PEG technique is the preferred approach in individuals with other indications. Family degree of satisfaction probably does not depend upon the technique itself if the most appropriate indication is performed, according to the patient medical history. These findings emphasize the future need for a unitary protocol in terms of safety methods for gastrostomy placement in children, considering age and underlying chronic illness.

## Figures and Tables

**Table 1:** General characterization of the subjects.

	Group 1 (Reflux Surgery)	Group 2 (No Reflux Surgery)	*p*-Value
No. of patients	34 (47.88)	37 (52.11)	1.33
Age (mean ± SD, years)	4.86 ± 1.09	5.15 ± 1.11	0.89
Sex (*n*, %)			
Male	14 (41.17)	20 (54.05)	0.78
Female	20 (58.82)	17 (45.94)	0.99
Area			
Urban	18 (52.94)	27 (72.97)	0.05
Rural	16 (47.05)	10 (27.02)	0.05
Indication (*n*, %)			
GERD	34 (100)	2 (5.40)	0.05
Malnutrition	3 (8.82)	6 (16.21)	0.05
Aspiration	2 (5.88)	5 (13.51)	0.05
Multiple	25 (50)	14 (37.83)	0.05
Genetic syndrome (*n*, %)	10 (29.41)	12 (43.43)	0.05
Aspiration pneumonia (*n*, %)	24 (70.58)	9 (24.32)	0.05
Z Weight (mean ± SD)	−2.33 ± 0.44	−2.76 ± 0.89	0.67
Z Height (mean ± SD)	−1.78 ± 0.56	−1.89 ± 0.97	1.32
Z score BMI	−3.11 ± 1.01	−3.23 ± 1.12	1.44
Previous feeding procedure			
Oral Naso-gastric tubes Mixed	5 (14.7) 20 (58.82) 9 (26.47)	9 (24.32) 26 (70.27) 3 (8.10)	0.79
Types of food used before GT			
Standard formula Enteral formula Normal diet Mixt	7 (20.58) 5 (14.7) 5 (14.7) 17 (50)	9 (24.32) 8 (21.62) 7 (18.91) 13 (35.13)	0.68
Meal duration in minutes (mean ± SD)	45 ± 12.33	45.72 ± 13.45	0.78
Partial oral nutrition Y/N (*n*, %)	5 (14.7)	11 (29.72)	0.05
Types of food used after GT			
Milk formula Home blenderized diet Modular diet	7 (20.58) 9 (26.47) 18 (52.94)	11 (29.72) 18 (48.64) 8 (21.62)	0.99
Time for the first feeding	8.10 ± 2.54	8.14 ± 2.11	0.88
Night continuous nutrition	1 (2.94)	0 (0)	NA

*n*—number; SD—standard deviation, NA—not applicable.

**Table 2:** Symptoms and complication variation.

	Group 1 (Reflux Surgery)	Group 2 (No Reflux Surgery)	*p*-Value
Symptoms			
Nausea (*n*, %)	3 (8.82)	5 (13.51)	0.05
Eructation (*n*, %)	1 (2.94)	3 (8.10)	0.05
Reflux (*n*, %)	1 (2.94)	1 (2.70)	0.12
Vomiting (*n*, %)	1 (2.94)	3 (8.10)	0.05
Flatulence (*n*, %)	3 (8.82)	5 (13.51)	0.05
abdominal pain (*n*, %)	10 (29.41)	9 (24.32)	0.99
Constipation (*n*, %)	2 (5.88)	2 (5.40)	0.76
Diarrhea (*n*, %)	0 (0)	0 (0)	NA
Mouth dryness (*n*, %)	1 (2.94)	0 (0)	NA
Complications			
Suppuration (*n*, %)	11 (32.35)	8 (21.62)	0.88
Infection (*n*, %)	2 (5.88)	1 (2.70)	0.67
Disjunction (*n*, %)	2 (5.88)	2 (5.40)	0.97
Fistula (*n*, %)	0 (0)	0 (0)	NA
Bumper retraction (*n*, %)	0 (0)	0 (0)	NA
Hospitalization duration (mean ± SD, days)	7.51 ± 3.56	4.22 ± 2.13	<0.005

*n*—number, SD—standard deviation, NA—not applicable.

**Table 3:** SAGA-8 dependent variable variation.

	SAGA-8		
	Yes	No	*p*-Value
Genetic syndrome	11.9	11.5	0.88
Aspiration pneumonia	14.3	12.3	0.05
Hospitalization > 5 days	12.9	13.6	0.76
Home blenderized diet	15.39	12.04	0.75

## Data Availability

The original contributions presented in this study are included in the article. Further inquiries can be directed to the corresponding author.

## Abbreviations

The following abbreviations are used in this manuscript:

ESPGHAN	European Society of Pediatric Gastroenterology Hepatology and Nutrition
PEG	Percutaneous endoscopic gastrostomy
GT	Gastrostomy tube
